# Spectroscopic profiling variations in extracellular vesicle biochemistry in a model of myogenesis

**DOI:** 10.1177/20417314211022092

**Published:** 2021-05-31

**Authors:** Owen G. Davies, Stephen Powell, Jonathan JS Rickard, Michael Clancy, Pola Goldberg Oppenheimer

**Affiliations:** 1 School of Sport, Exercise and Health Sciences, Loughborough University, Loughborough, UK; 2 School of Chemical Engineering, University of Birmingham, Birmingham, UK; 3 Physical Sciences for Health Doctoral Training Centre, University of Birmingham, Birmingham, UK; 4 Department of Physics, Cavendish Laboratories, University of Cambridge, Cambridge, UK

**Keywords:** Vesicle, skeletal muscle, differentiation, Raman spectroscopy, analytical

## Abstract

Extracellular vesicles (EVs) hold value as accessible biomarkers for understanding cellular differentiation and related pathologies. Herein, EV biomarkers in models of skeletal muscle dormancy and differentiation have been comparatively profiled using Raman spectroscopy (RS). Significant variations in the biochemical fingerprint of EVs were detected, with an elevation in peaks associated with lipid and protein signatures during early myogenic differentiation (day 2). Principal component analysis revealed a clear separation between the spectra of EVs derived from myogenic and senescent cell types, with non-overlapping interquartile ranges and population median. Observations aligned with nanoparticle tracking data, highlighting a significant early reduction in EV concentration in senescent myoblast cultures as well as notable variations in EV morphology and diameter. As differentiation progressed physical and biochemical differences in the properties of EVs became less pronounced. This study demonstrates the applicability of RS as a high-resolution analytical method for profiling biochemical changes in EVs during early myogenesis.

## Introduction

We live in an ageing society, with the number of individuals over 60 predicted to nearly double by 2050.^1^ The ageing phenotype is heterogeneous, with individuals of the same chronological age differing considerably in their health status. Consequently, considerable attention is being focused on defining biological markers that can predict the onset of age-related diseases and allow for timely clinical intervention. Sarcopenia is defined as an age-related loss of muscle mass, strength and function. A loss of muscle mass typically begins at age 40, when individuals start to lose 1%–2% of muscle per year. By the time an individual reaches age 70 they have typically lost 25%–30% of their total muscle mass, with a corresponding decrease in muscle strength of up to 40%.^2^ The etiology of sarcopenia is complex and incompletely understood, with micro- and macro-structural changes in muscle anatomy observed due to age-related variations in muscle metabolism, proteostasis and cell senescence.^3^ Senescence is a well-documented component of age-related diseases, with accumulating evidence linking a senescence associated secretory phenotype (SASP) to chronic impairments in stem cell function and the attenuation of skeletal muscle regeneration during ageing.^4^ Thus, understanding and targeting cellular senescence during skeletal muscle ageing could have implications for the monitoring and treatment of sarcopenia.

The multidimensionality of the sarcopenic process means that single biomarker approaches are likely insufficient to capture the underlying complexity of how SASP impacts healthy ageing. In this regard, naturally synthesised biological nanoparticles termed extracellular vesicles (EVs) are appealing since they are ubiquitous, accessible and contain a complex and varied cargo that is likely to be cell and disease specific. Currently little is known about the contribution of EVs to myoblast senescence and how this impacts on muscle ageing.^5^ To date, studies have shown that myoblasts (MBs) and myotubes (MTs) release EVs rich in RNA, mtDNA and proteins that are selectively sorted and have modulatory effects on myogenesis and muscle development.^6,7^ An observation by Choi et al.^8^ demonstrated that EVs derived from differentiating human skeletal MBs contained multiple myogenic growth factors (e.g. insulin-like growth factors, fibroblast growth factor-2) and led to an increased number of regenerated myofibres in a murine muscle laceration model. Recent studies have also documented variations in the concentration of circulating plasma-derived small EVs in older adults exhibiting physical frailty and sarcopenia.^9^ However, this heterogenous fraction is likely to contain EVs derived from multiple diverse cellular sources (e.g. circulating white blood cells) and does not specifically identify local changes in skeletal muscle physiology. Acknowledging this heterogeneity, attempts have been made to map specific variations in circulating skeletal muscle-derived EVs through the analysis of EVs expressing α-sarcoglycan (SGCA) and the muscle-specific microRNA miR-206, with increases in the SGCA^+^ fraction observed following acute exercise. However, these SGCA^+^ EVs accounted for only between 1% and 5% of the total population.^10^ In addition to the identification of circulating skeletal muscle-derived EVs, there is evidence for the role of electron-dense vesicles at sites of MB fusion, where they are thought to deliver lipids and adhesion proteins important for membrane union.^11^ This collective evidence has begun to identify potentially diverse functions of skeletal muscle EVs in local and systemic events related to muscle physiology. However, the impact of cellular senescence on EVs localized within the secretome remains undefined.

To date, studies have analysed the protein and RNA content of MB EVs using mass spectrometry and real-time PCR in an attempt to identify disease-specific molecules.^12,13^ These methods are critical in advancing our fundamental understanding of many pathologies but are often too costly, complex and time-consuming to be applied for routine analysis. As such, sensitive bioanalytical methods that allow for high-throughput, accurate and label-free analysis are required. Raman spectroscopy (RS) is a label-free, non-invasive and highly sensitive real-time method. This technique provides both qualitative and quantitative determination of the chemical composition from just a limited volume of sample (typically a few microliters). It eliminates the requirement for specific protein biomarkers, rendering it a promising tool for the detection of unique fingerprints in complex multifactorial pathologies. To date, studies have shown that RS can be applied to cytologically distinguish EVs originating from a variety of cell types. For example, Gualerzi et al.,^14^ distinguished tissue-specific biochemical signatures (primarily in the lipid component) between mesenchymal stromal cells derived from adipose tissue, bone marrow and dermal fibroblasts.

A growing number of publications have also demonstrated the utility of RS to differentiate EVs derived from cancerous and non-cancerous cell types.^15^ These important findings are driving the development of portable diagnostic Raman assays that may one day have utility in a clinical setting.^16^ Based on these findings we aimed to provide the first example of RS as a method to distinguish between EVs generated by actively differentiating MBs and senescent cultures where limited myogenesis was observed. Through the application of this method, we provide a fundamental insight into how EV profiles change within the secretome of senescent MBs associated with skeletal muscle ageing. In the study we applied Raman spectroscopy to generate the very first profile of variations in potential EV biomarkers during early MB fusion events. This is the first time this label-free approach has been applied to study myogenesis. Such an approach could provide potential targets that can be applied for the profiling of comparatively complex biofluids such as, blood plasma to begin to identify pathological age-related variations in the regenerative capacity of skeletal muscle.

## Materials and methods

### Cell culture

Fetal bovine serum (FBS, Sigma-Aldrich, UK) used throughout the study was depleted of EVs by ultracentrifugation at 120,000 xg for 18 h. C2C12 immortalised murine MBs (CRL-1772; ATCC, Rockville, MD, USA) were cultured in high glucose DMEM supplemented with 20% FBS and 1% penicillin/streptomycin (Sigma-Aldrich, UK). To induce myogenic differentiation the medium was replaced with high glucose DMEM supplemented with 1% horse serum (Sigma-Aldrich, UK) and 1% penicillin/streptomycin (Sigma-Aldrich, UK).

### Generation of non-differentiating senescent myoblasts

An original stock of C2C12 cells was seeded at a density of 26 × 10^3^ cells per cm^2^ and maintained at 37°C/5% CO_2_ in growth medium. Upon reaching confluence the medium was substituted for myogenic medium and the cultures differentiated for a period of 6 days until mature myotubes were evident. Cultures were then trypsinised, centrifuged at 300*g* and re-plated to isolate unfused myoblasts. MTs do not re-adhere and are removed during the first medium change. This process was repeated five times until the presence of MTs could no longer be observed.

### Fluorescence microscopy

In accordance with a previously published protocol.^17^ Samples were washed with 1% Tris-buffered saline (TBS) and fixed using methanol–acetone (1:1) (Sigma-Aldrich, UK). Samples were washed and subsequently immersed in 500 μl blocking solution [5% goat serum (Gibco, UK) and 0.2% triton-X (Sigma-Aldrich, UK) in TBS]. After the blocking step, each well was incubated with rhodamine phalloidin (1:500, Fisher) for 1 h to visualize the actin cytoskeleton. 4ʹ,6-diamidino-2-phenylindole (DAPI; Sigma-Aldrich, UK) was added for a period of 10 min to visualize nuclei. Images were recorded using a Leica DM2500 fluorescence microscope (Leica, Cambridge, UK). Scale measurements were generated using ImageJ software.

### Extracellular vesicle isolation

Myogenic and senescent C2C12 myoblasts were cultured at scale in T175 flaks (Nunc, UK) over a total period of 13 days. EVs were isolated from culture medium at day 2, 6, 10 and 13 by differential centrifugation as outlined in a previous publication.^18^ Samples were centrifuged at 2000*g* for 20 min to remove cell debris and apoptotic bodies, 10,000*g* for 30 min to remove larger micro-vesicles, 120,000*g* for 70 min to pellet EVs. Following the final ultracentrifugation step, the supernatant was removed, the pellet washed in sterile 0.2 μm filtered phosphate buffered saline (PBS) and further centrifuged at 120,000*g*. All ultracentrifugation steps were performed using a Sorvall WX Ultra Series Ultracentrifuge (Thermo Scientific, UK) with a Fiberlite, F50L-8X39 fixed-angle rotor (Piramoon Technologies Inc., USA). The resulting pellet was re-suspended in 300 μL of PBS and stored at −80°C for analysis.

### Dynamic light scattering (DLS)

Particle size distribution and intensity was analysed using Dynamic Light Scattering (DLS) using a ZetaSizer 3000-HA (Malvern Instruments, UK). Particles were diluted 1:100 using sterile filtered PBS and analysed using settings recommended by the supplier (refractive index = 1.39, viscosity = 0.89, absorption = 0.01).

### Nanoparticle tracking analysis (NTA)

EV size and concentration was measured using a Malvern NanoSight LM10 instrument (Malvern Instruments, UK). The camera level was set to 10 and the detection threshold and gain maintained at 14 and 3, respectively. Videos were captured for a period of 60 s at 30 frames per second using NTA 3.1 software.

### CD63 enzyme-linked immunosorbent assay (ELISA)

The tetraspanin marker CD63 was used to provide a quantitative measure of EV number. CD63 levels were quantified using the ExoELISA-ULTRA kit (System Bioscience, UK). Equal volumes of EVs samples were immobilised onto the wells of a microtitre plate and the assay carried out according to the manufacturer’s instructions.

### Transmission electron microscopy

A suspension of EVs was made in sterile PBS. A single drop of the suspension (~20 μL) was deposited onto a carbon film grid (Agar Scientific, UK) and left to air dry for a period of 1 min. To remove excess PBS the grid was blotted using tissue paper through capillary action. Negative staining was achieved by placing a single drop (~20 μL) of uranyl acetate solution onto the dry grid. Samples were imaged using a transmission electron microscope (FEI Philips Tecnai 20) using a voltage of 80kV.^18^

### Western blotting

The presence of EV associated markers was confirmed as outlined in a previous publication.^19^ Following electrophoretic separation using precast 4%–15% Mini-PROTEAN TGX gels (Biorad), the gels were blotted onto polyvinylidene difluoride (PVDF) membranes (Invitrogen) and blocked with 5% non-fat milk powder in Tris-buffered saline. After 1 h blocking at room temperature, the membranes were incubated with primary antibodies against Annexin A2 (37 kDa, Abcam, UK) and Alix (97 kDa, Santa Cruz, USA) in their respective blocking buffers overnight at 4 °C at a dilution of 1:1000 in Tris-buffered saline containing 0.5% Triton X 100. After washing, blots were incubated with 1:3000 horseradish peroxidase-conjugated secondary antibodies (Cell Signaling, UK) for 1 h at room temperature. Chemiluminescence detection of bands was performed with ECL Plus reagent (Biorad) and imaged using Image Lab Software (Life Science Research, BioRad, UK).

### Confocal Raman spectroscopy

Raman spectroscopy measurements were carried out with an InVia Qontor Renishaw Raman Microscope System equipped with a 785 nm laser. The spectra were typically acquired with a 10 s exposure time and a laser power of 10 mW at the sample. A 50× objective with a numerical aperture of 0.75 was used for all measurements. 10 µl of EV solution was deposited on the coated slide and left to air dry before analysis. Raman spectra were obtained following the protocol established by Gualerzi et al.^20^ For each time point there were six EV samples processed for both differentiating and senescent myoblasts, with a minimum of three spectra per sample (*n* > 18). Spectra were subject to baseline correction followed by feature scaling normalisation (between 0 and 1) before statistical analysis in MATLAB. The data was mean centered *prior* to the principal component analysis in MATLAB. Average spectra were subject to third order polynomial smoothing with a 9-point window. Optical measurements were carried out with Leica (DM2500) microscope equipped with an incoherent white light (halogen) source and an optical fibre coupled to a QE65000 Ocean Optics spectrometer. The spectra were normalized with respect to those recorded on flat silicon.

### Statistical analysis

Values are show as mean ± standard deviation. Student’s T tests were performed using Microsoft Excel and analysis of variance (ANOVA) with Bonferroni *post-hoc* using MiniTAB® v.16 (Minitab Inc., State College, PA, USA). Wilcoxon rank-sum tests were performed in MATLAB 2018a along with the principal component analysis.

## Results and discussion

**Figure 1.**
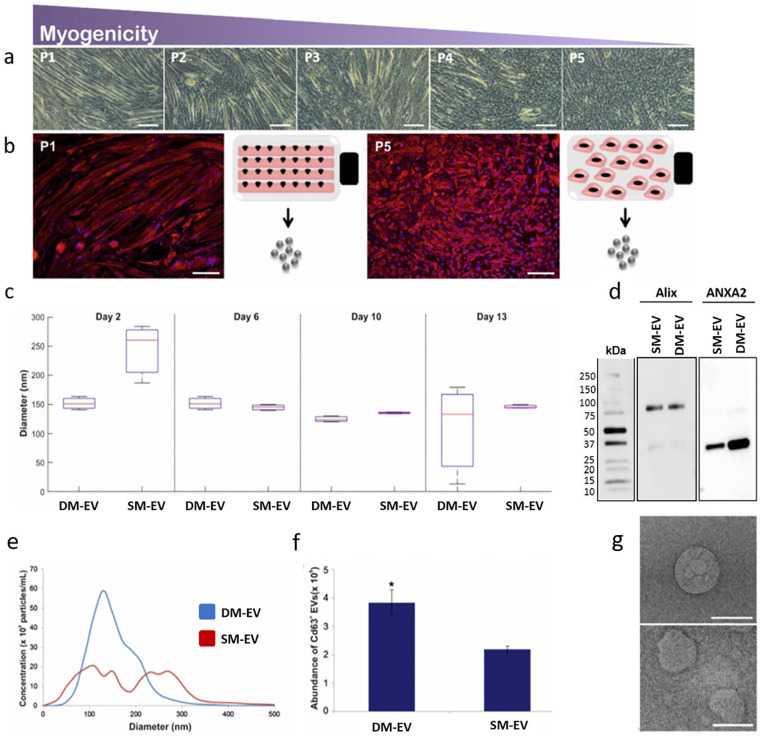
The isolation and profiling of EVs isolated from DM and SMs: (a) optical micrographs displaying a progressive reduction in myogenesis during five rounds of selection for SMs (P1 to P5), (b) fluorescence microscopy images highlight changes in the cytoskeletal arrangement of actin in (P1) and SM (P5) that evidence limited myotube formation in the latter, scale bars represent 100 μm, (c) comparative dynamic light scattering (DLS) analysis of vesicle diameter over a period of 13 days myogenic culture, (d) western blotting for common EV markers, Alix and ANAXA2. Samples were run on the same gel, with 5 µg protein loaded for each. Length of exposure was adjusted for each protein at the time of image acquisition, (e) nanoparticle particle tracking (NTA) analysis confirming initial differences in EV diameter between DM-EVs and SM-EVs after two days in myogenic medium, (f) enzyme-linked immunosorbent assay (ELISA) for the EV tetraspanin marker CD63 displaying significant differences in the concentration of CD63^+^ EVs isolated from DM (3.84 × 10^9^) and SM (2.19 × 10^9^), and (g) transmission electron micrographs of DM-EVs (top) and SM-EVs (bottom). Scale bars 200 nm.

C2C12 MBs were cultured in myogenic differentiation medium until mature MTs were observed. A detailed description of methods applied is included in the ‘Materials and Methods’ section. C2C12s that had not undergone fusion were isolated using an established protocol and expanded through additional cycles of differentiation/isolation to generate a pool of senescent myoblasts (SM) with limited myogenic capacity that is representative of age-related senescence (Figure 1(a) and (b)).^17,21^ EVs derived from actively differentiating MBs (DM, P1) and senescent cells (SCs, P5) were compared to define whether fundamental biochemical differences at the nanoscale correlated with an observed reduction in myogenic capacity. Culture medium was recovered over a period of 13 days and EVs isolated. Comparisons revealed an increase in the size of EVs derived from SM (SM-EVs), perhaps indicating a shift between exosome (30–150 nm) and microvesicle (50–1000 nm) biogenesis (Figure 1(c)).^22^ Based on the EV isolation method employed in this study, we can exclude the presence of comparatively dense large apoptotic bodies from our EV fractions (removed via a 30 min centrifugation at 10,000*g*), we acknowledge that apoptotic cell-derived extracellular vesicles (ApoEVs) that share a common size, density and marker profile with traditional EVs will be retained in the EV preparation and further studies are required to understand the possible function of ApoEVs in skeletal muscle ageing.^23^ At subsequent time points, the median size of EVs from both DM and SM cultures did not vary significantly. However, considerable variation in the particle sizes of DM-EVs was evident at day 13. Further analysis at day 2 using nanoparticle tracking analysis (NTA) identified that SM EV displayed a bimodal distribution, with distinct peaks likely representative of exosomes and microvesicles present at 110 nm and 270 nm, respectively (Figure 1(d)). In comparison, DM-EVs were unimodal with a median size of 130 nm with no evidence of microvesicle-sized particles (Figure 1(d)). Quantification of CD63 revealed a significant (*p* < 0.05) reduction in the concentration of SM-EVs relative to DM-EVs, with values of 2.19 × 10^9^ and 3.84 × 10^9^ respectively (Figure 1(e)). Both samples were positive for additional EV marker proteins (Alix and ANAXA2), with visual evidence of an increase in ANAXA2 in DM-EVs (Figure 1(f). Heterogeneity within SM-EV samples were visually evidenced using transmission electron microscopy, with evidence of membrane deformation when compared with DM-EVs (Figure 1(g)). Although both DM and SMs were cultured under standard myogenic conditions (with no fusion evident in SM cultures), it remains to be determined whether the respective differences observed in EV profile are reflective of variations in EV biogenesis that have been proposed to result between MBs and MTs, with levels of the endosomal marker Alix previously found to be increased in MTs.^24^ Outcomes from the present study identified no obvious qualitative differences in Alix between EVs isolated from DM and SM cultures. However, significant differences were observed in the presence of CD63^+^ particles, which could suggest that DMs release a higher proportion of exosome-like particles when compared with SM cultures. However, we acknowledge that CD63 is not universally expressed on all exosome-like particles and we identify a need for further research into defining the precise biogenesis and function of EVs during the myogenic process.^25^ We also identify a need to further profile possible heterogeneity within the exosome-like fraction through the application of density gradient centrifugation, which would allow for the characterization of subpopulations based on density.^26^

**Figure 2.**
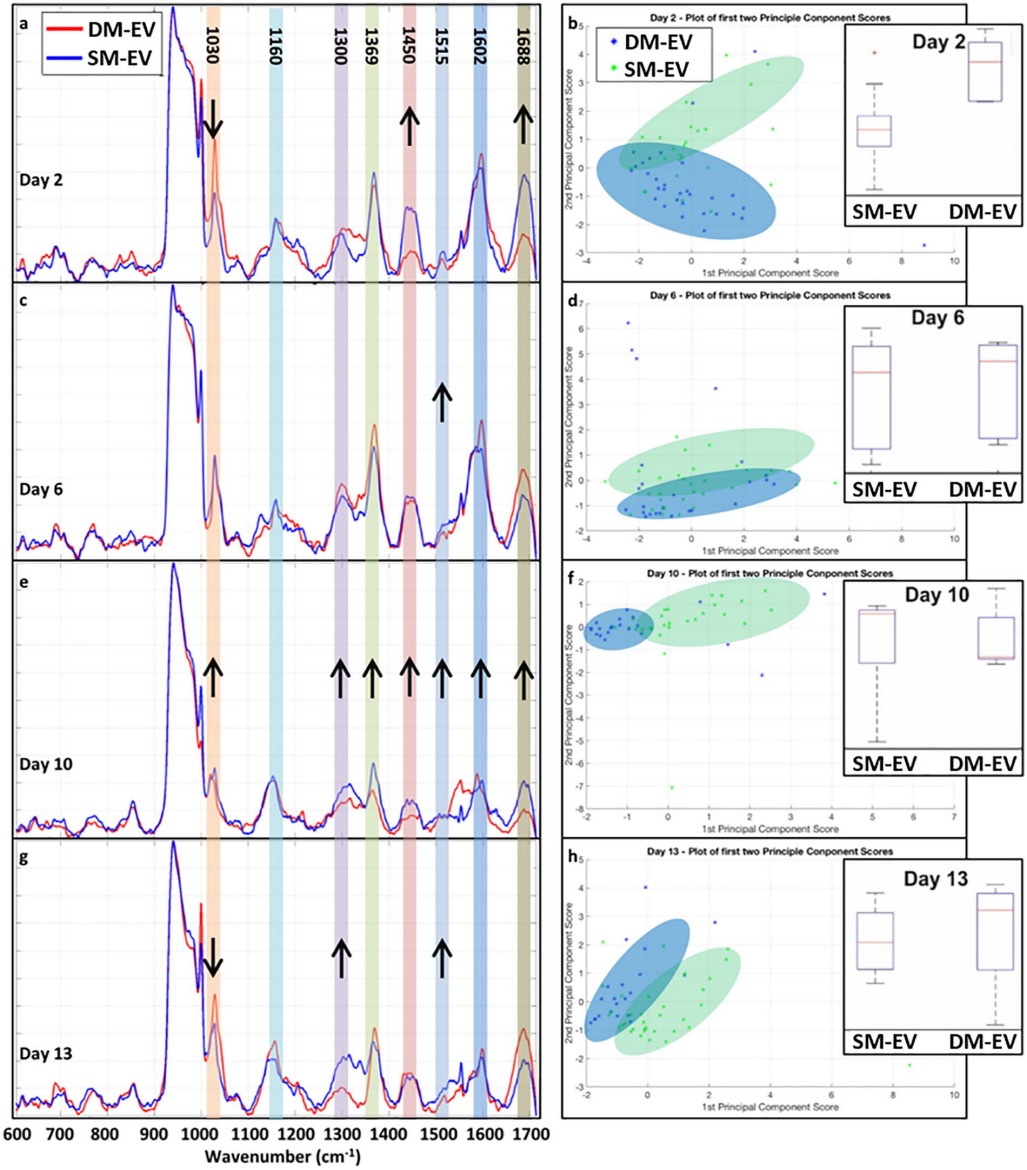
Average Raman spectra DM-EVs (blue) and SM-EVs (red), shown alongside the PCA scores and the boxplots (insets) of the scores from the second principal components of each cell line. In the boxplots, the interquartile range is shown by the blue box, the population median is shown by the red line, the whiskers on the boxes show the tails of the distribution and the red dots show population outliers: (a) averaged spectra at day 2, (b) second principal component for day 2, (c) averaged spectra at day 6, (d) second principal component for day 6, (e) averaged spectra at day 10, (f) second principal component for day 10, (g) averaged spectra at day 13, and (h) second principal component for day 13.

Using Raman spectroscopy, we identified distinct fingerprints for DM-EVs and SM-EVs over a period of 13 days cultured in myogenic medium (Figure 2(a)–(d)). The most prominent Raman peaks were associated with changes in lipids, proteins and the putative amide I and III bands at 1030, 1160, 1300, 1369, 1450, 1515, 1600 and 1688 cm^-1^.^27–34^ Changes in these peaks were analysed using a Wilcoxon rank-sum test to determine any statistically significant peak changes between DM-EVs (used as a control) and SM-EVs. *p*-values less than 0.01 were considered significant. PCA analysis of the day 2 results showed clear separation of the DM-EV (*n* = 31) and SM-EV (*n* = 25) spectra with non-overlapping interquartile ranges and population medians in the second component (Figure 2(b)). This was confirmed by the rank-sum test, which indicated an increase in protein peaks 1450 (due to the CH_2_/CH_3_ deformation vibrations) and 1688 cm^−1^ (*p*-values < 0.01) and a decrease in the 1030 cm^−1^ protein peak from the DM-EV to SM-EV spectra. At day 6, despite the average spectra showing differences, the only significant change was found in the 1515 cm^−1^ protein peak (*p*-value < 0.01), which showed an increase in the SM-EV (*n* = 32) spectra compared to DM-EVs (*n* = 25). This is supported by the PCA analysis, which showed overlap in the DM-EV and SM-EV interquartile ranges in the second component, indicating the variation between cell types is hard to distinguish from variation between samples (Figure 2(c) and (d)).

Significant increases (*p*-values < 0.01) in the intensity of all peaks, other than 1160 cm^−1^, were found at day 10 for SM-EVs (*n* = 28) compared to DM-EVs (*n* = 22) (Figure 2(e)). As with day 2, the increase in the 1450 cm^−1^ band correlates with the increase in the 1688 cm^−1^ protein band. This indicated an increase in proteins and lipid concentration compared to the control group. Again, this is supported by the PCA analysis (Figure 2(f)) which showed ‘skewed’ population medians in the interquartile ranges of the second component, indicating that there were distinct variations in EV composition. The PC1-PC2 scatter plots on day 10 also show a clear separation in the first component, not seen at any other time point, which is supported by the statistically significant changes in the key peak intensities. On day 13 for the SM-EV samples (*n* = 34), there was a decrease in the 1030 cm^−1^ protein peak intensity (as seen at day 2) from the DM-EV controls (*n* = 21) and also an increase in the 1300 cm^−1^ lipid peak and the 1515 cm^−1^ protein peak (*p*-values < 0.01) (Figure 2(g)). This variation between the two groups was also seen in the PCA data (Figure 2(h)), where although we saw overlap of the distributions in the first component, in the second component the distributions showed separation. PCA of all Raman spectra combined from day 2, 6, 10 and 13 was also performed. This has provided no additional insight into the data and therefore was omitted for the final analyses (Supplemental Figure S1). The EVs data was further analysed through a self-organising map (SOM) classification model implementing learn vector quantisation (LVQ),^35^ (1000 learning step and a learning rate of 0.5). The SOM data supports the PCA findings in terms of being able to distinguish between the two EV populations at Days 2, 10 and 13 but not at day 6 (Supplemental Figure S2). As an additional metric, a lipid-to-protein ratio was calculated by adapting the protocols used by Mihály et al.,^20^ and Gualerzi et al.,^36^ for IR and RS respectively. The previous protocols used high wavenumber peaks (2750–3040 cm^−1^) for lipids and 1600–1690 cm^−1^ for proteins. However, as high wavenumber data was not collected, the 1300 cm^−1^ peak was used as the lipid reference and the 1688 cm^−1^ for the protein reference. Analysis of the lipid-protein ratio across all samples and time points, using a Wilcoxon rank-sum test, showed that at day 2 there was a significant decrease in the lipid-to-protein ratio from the DM-EV controls to the SM-EV samples. At days 6 and 10, despite several significant changes in peak intensity between the DM-EV and SM-EV spectra, there was no significant change in the lipid-to-protein ratio. At day 13, we saw a shift in the direction of the lipid-to-protein ratio as there was a significant increase from the DM-EV controls to the SM-EV samples, indicating an increase in lipid concentration over protein.

RS has shown considerable utility as a label-free and non-invasive method for monitoring regeneration in musculoskeletal tissues such as bone and tendon. However, its application in skeletal muscle remains relatively unexplored. Through the application of RS, we identified temporal differences in the protein and lipid content of EVs derived from SM and DM cultures. Differences were most significant at day 2 and correlated with limited myogenic fusion, suggesting that initial early biochemical variations in EV composition could influence cell fusion events required for myogenesis. These findings are highly relevant given that GFP-labelled vesicles generated by MTs have been shown to become internalised by non-fluorescent MBs, indicating active EV uptake as a mechanism for driving myogenesis.^12^ Furthermore, EVs have previously been found to contain important fusogenic proteins such as integrin β1 and cadherin-13, with cadherin-13 upregulated during differentiation.^37^ Further, prominent proteins associated with lipid domains, such as N-cadherin have also previously been localised to secretory and endocytic vesicles derived from the Golgi apparatus and have demonstrated downstream effects on the expression of essential myogenic genes.^38^ Consequently, the present study suggests that particular focus needs to be applied to understanding the composition and role of EVs generated at the initiation of myogenesis, since biochemical variations in these particles correlate with a decline in MB fusion.

In addition to myogenic proteins, membrane lipid composition is highly important for coordinating fusion events critical to skeletal muscle regeneration.^39^ Through the application of RS, Muratore et al.,^40^ distinguished the membranes of C2C12 MBs are comparatively enriched in saturated lipids (1450 cm^−1^) when compared with fibroblasts, which increases membrane capacitance required for electrophysiological stimulation. Further, it has been long acknowledged that alterations in membrane fatty acyl composition can modulate the fusion of MBs, with acyl chains enriched in elaidate or the polar headgroups of phosphatidylethanolamine (PE) found to inhibit fusion, while those enriched in oleate enhanced fusion.^39^ In alignment with these findings, we distinguished a significant reduction in the 1450 cm^−1^ peak of SM-EVs, which could highlight the utility of RS to distinguish variations in EV saturated lipids and provide a rapid, label-free indication of myogenic capacity. In addition to RS studies on membrane lipids, Ichimura et al.,^41^ applied RS to assess biochemical changes associated with C2C12 myogenesis identifying localised variations in cytosolic protein and lipid content 3 days after myogenic induction. In the present study we identified significant changes in EV lipid and protein content just 2 days after myogenic induction, which may further highlight the value of EVs as early predictive indicators of myogenesis. Unlike the study by Ichimura et al., we did not observe any significant spectral changes at 753 cm^1^, which the authors attribute to cytochrome C and the apoptosis of cells that do not undergo myogenic differentiation. This could perhaps be explained by the fact the EV isolation protocol employed in our study eliminated larger particles such as apoptotic bodies. Consequently, changes in EV lipid composition could indirectly affect myogenic fusion via a reduced interaction with prominent cell surface proteins and negatively impact molecular events underlying for muscle regeneration.

Finally, it is worth mentioning that no ‘standard’ Raman spectra for EVs yet exists, with the majority of studies focusing on heterogeneous populations of particles isolated from varying tissue and biofluid sources and implicated in different biological processes and a range of associated diseases. This is further exacerbated by the differences in spectra likely introduced by the multitude of available EV isolation protocols applied throughout the literature. For example, the ratio of amide I protein band to the lipid related bands has been shown to vary in samples depending on the isolation protocols of EVs.^20^ It is widely appreciated that isolation methods such as size-exclusion chromatography (SEC) can result in the recovery of lipoproteins that will impact downstream Raman analysis. While variations to standard UC protocols seen throughout the literature will undoubtedly influence the EV recovery and reproducibility of studies. Further differences are likely to result from the parental cell type and biogenesis/size of EVs isolated – with some methods applying cut-offs in an attempt to isolate only exosome- or microvesicle-like particles.^14,28,42^ In the present study we applied a standard UC protocol without cut-offs in an unbiased attempt to isolate a broad range of EVs and provide a broad overview of biochemical changes. Since our study is the first to apply RS to profile EVs derived from skeletal muscle myoblasts, we strongly believe that this protocol provides the most general and informative overview of spectral changes occurring during myogenesis and serves as an appropriate benchmark for further studies.

## Conclusions

In conclusion, our findings provide a proof-of-concept demonstration of the application of RS to detect chemical variations in EVs indicative of in-vitro myogenic potential. Through the application of this method, we have provided important evidence to demonstrate that the biochemical fingerprint of accessible biological EVs can provide a label-free, non-invasive and quantifiable measure of some the earliest cell fusion events governing muscle development and regeneration. The study provides an initial benchmark for the in-vivo validation of these outcomes, providing potential targets that can be taken forward to identify age-related variations in basic skeletal muscle development.

## Supplemental Material

sj-pdf-1-tej-10.1177_20417314211022092 – Supplemental material for Spectroscopic profiling variations in extracellular vesicle biochemistry in a model of myogenesis Click here for additional data file. Supplemental material, sj-pdf-1-tej-10.1177_20417314211022092 for Spectroscopic profiling variations in extracellular vesicle biochemistry in a model of myogenesis by Owen G. Davies, Stephen Powell, Jonathan JS Rickard, Michael Clancy and Pola Goldberg Oppenheimer in Journal of Tissue Engineering
