# Supplementary material for: Spectroscopic profiling variations in extracellular vesicle biochemistry in a model of myogenesis
Source: J Tissue Eng. 2021 May 31;12:20417314211022092. doi: 10.1177/20417314211022092 (PMC8172953; doi:10.1177/20417314211022092)
Supplement: sj-pdf-1-tej-10.1177_20417314211022092 – Supplemental material for Spectroscopic profiling variations in extracellular vesicle biochemistry in a model of myogenesis [file sj-pdf-1-tej-10.1177_20417314211022092.pdf]

# - SUPPORTING INFORMATION -

Spectroscopic Profiling Variations in Extracellular Vesicle Biochemistry in a Model of Myogenesis

Owen G. Davies [a,c,], Stephen Powell [b], Jonathan J. S. Rickard [d], Michael Clancy [c], Pola Goldberg Oppenheimer [c,**]*

[a] School of Sport, Exercise and Health Sciences, Loughborough University, LE11 3TU, UK.
[b] Physical Sciences for Health Doctoral Training Centre, University of Birmingham, B15 2TT, UK.
[c] School of Chemical Engineering, University of Birmingham, B15 2TT, UK.
[d] Department of Physics, Cavendish Laboratories, University of Cambridge, CB3 0HE, UK.

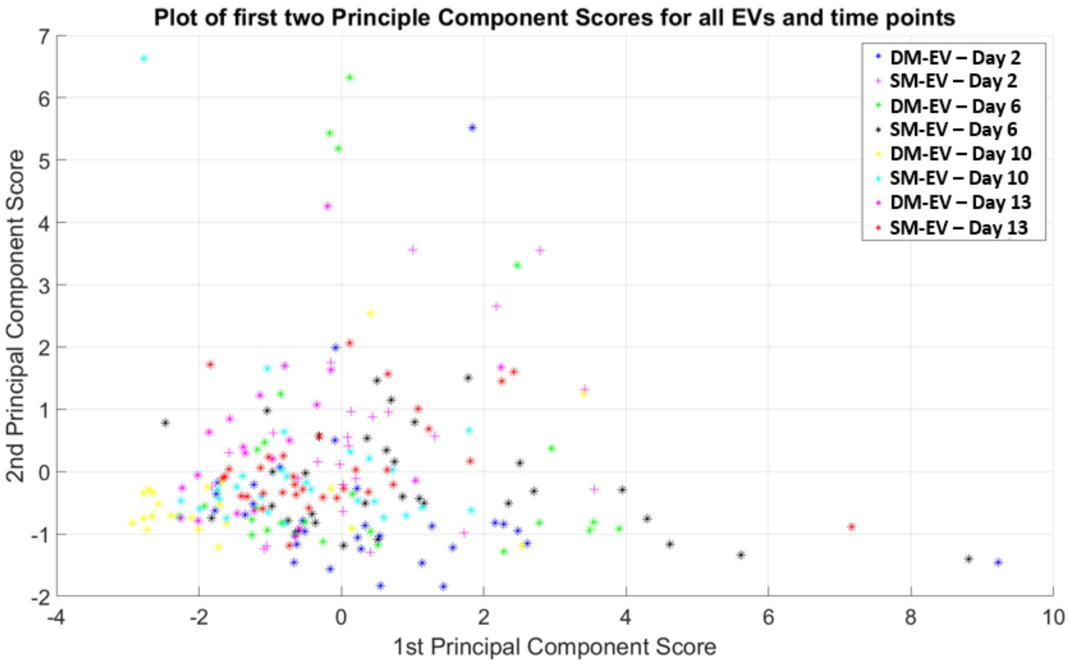

Figure S1. PCA of all Raman spectra combined from day 2, 6, 10 and 13 was also performed. This has provided no additional insight into the data and therefore was omitted for the final analyses.

The EVs data was further analysed through a self-organising map (SOM) classification model implementing learn vector quantisation (LVQ),[1] (1000 learning step and a learning rate of 0.5). The SOM data supports the PCA findings in terms of being able to distinguish between the two EV populations at Days 2, 10 and 13 but not at day 6.

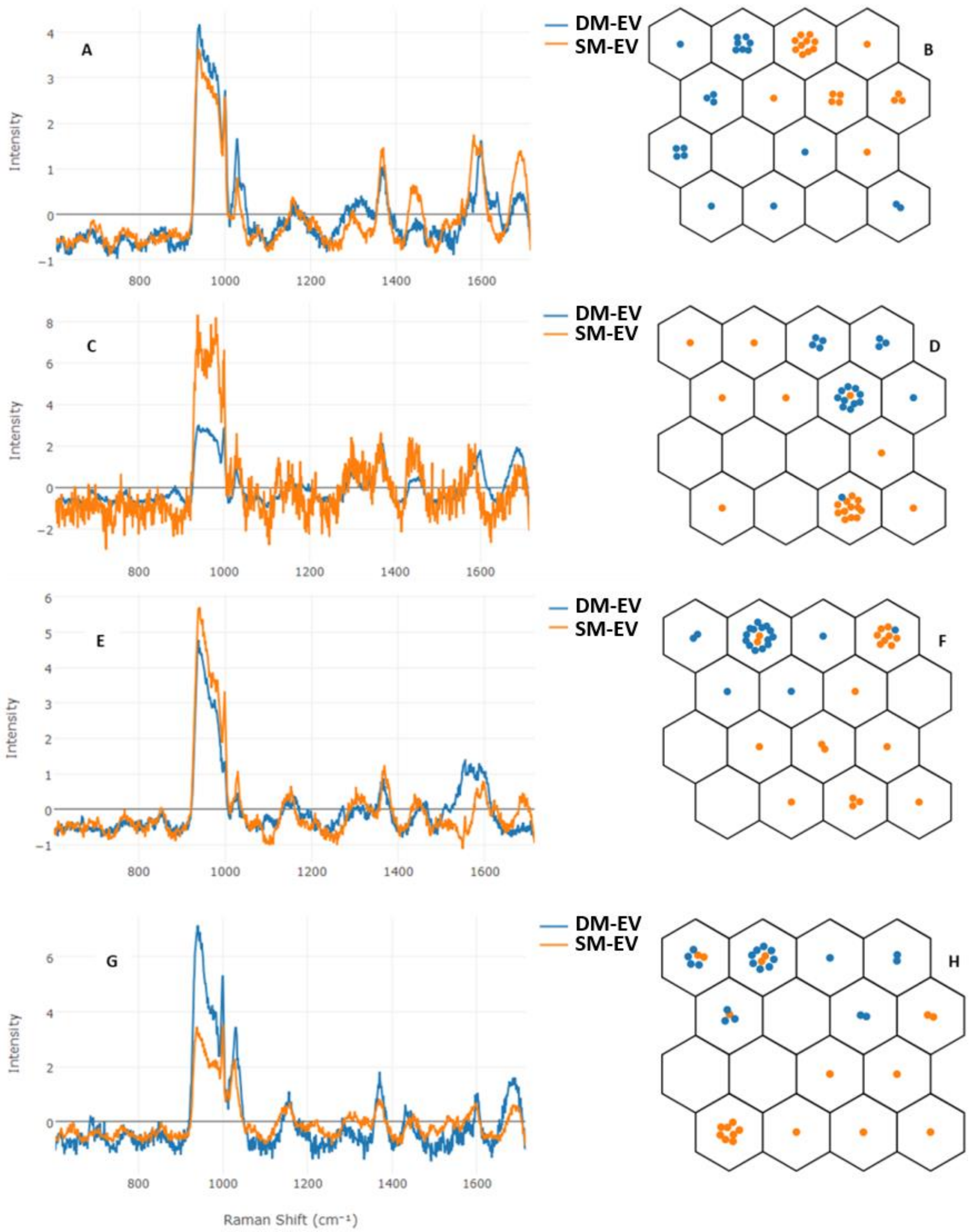

Figure S2. At day 2 the SOM classification showed a clear separation of the two EV groups and a classification accuracy of 93.75% from 16 test samples (**S3, b**). **Figure a** shows the plots of the SOM discriminant index, which highlights the peaks which contribute the most to the classification of the samples. This plot supports the statistical analysis of the peaks in the average spectra in showing clear differences between the EV groups around the peaks at 1030, 1450 and 1688cm^{-1}. **Figures c-d** show the SOM results for day 6 and despite appearing to separate the EV groups, the SOM only obtained 50% classification accuracy from 18 test samples suggesting a high variance across the test and training data as seen in the PCA results. The SOM results from Day 10 (**Figures e-f**) also showed a good separation between the EV groups, obtaining 81.82% classification accuracy from 11 samples. The SOM discriminant index plot also supports the peak analysis findings, indicating changes across all the key peaks. The SOM classification from day 13 again showed a separation in the two EV groups and achieved 73% classification accuracy from 15 test samples.

[1]. Development of the Self-Optimising Kohonen Index Network (SKiNET) for Raman Spectroscopy Based Detection of Anatomical Eye Tissue. Banbury, C., Mason, R., Styles, I., Eisenstein, N., Clancy, M., Belli, A., Logan, A., Goldberg Oppenheimer, P. *Scientific Reports*, **2019**, 9, 10812.

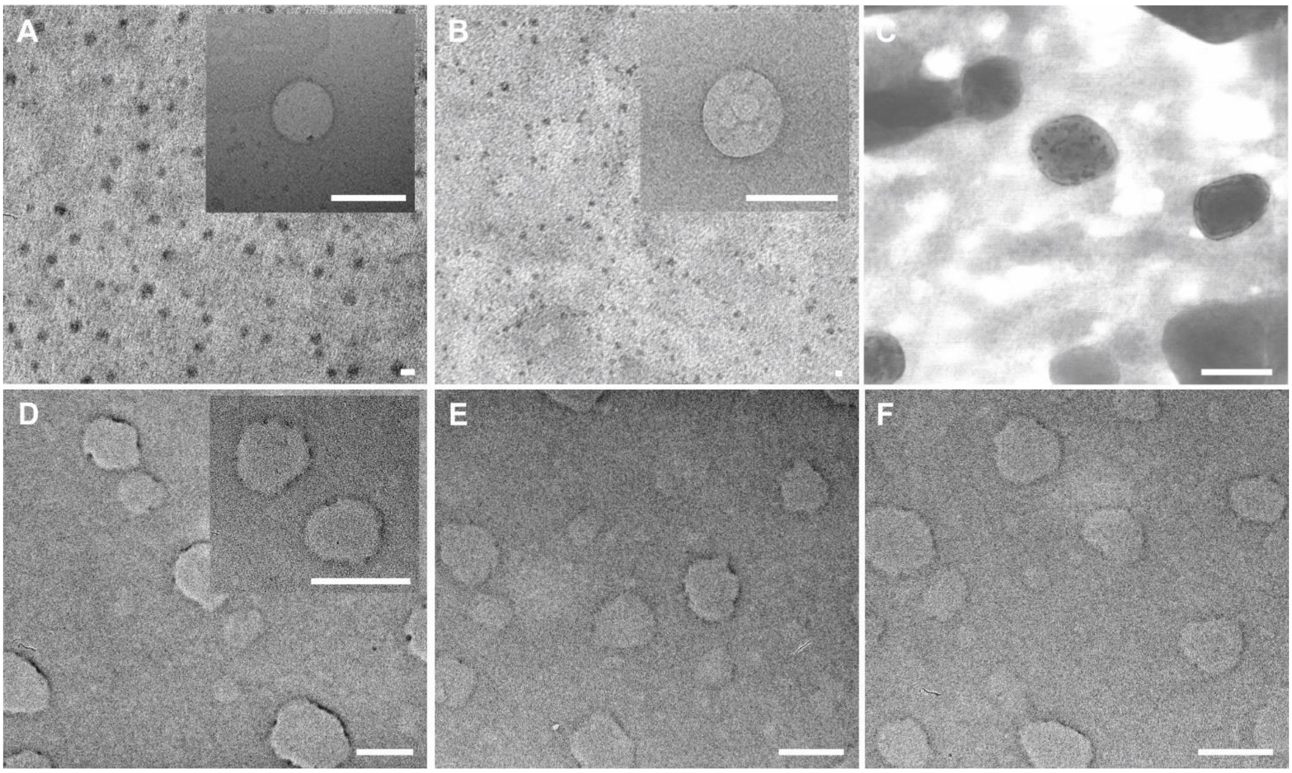

Figure S3. Transmission electron micrographs of **(a-c)** DM-EVs on **(a)** day2, **(b and b, inset)** day 6 and 10 and **(c)** day 13 and TEM images of SM-EVs on **(d)** day 2, **(d inset)** day 6, **(e)** day 10 and **(f)** day 13, accordingly. Scale bar: 200nm.
